# Supplementary material for: De novo design of a homo-trimeric amantadine-binding protein
Source: eLife. 2019 Dec 19;8:e47839. doi: 10.7554/eLife.47839 (PMC6922598; doi:10.7554/eLife.47839)
Supplement: Supplementary file 1. — Sample RosettaScripts XML file Supplementary file 1B Parameter constraint file for amantadine (.cst). Parameter constraint file for amantadine used in the RosettaDesign calculations. Supplementary file 1C Parameter definition file for amantadine (.params). Parameter definition file for amantadine used in the RosettaDesign calculations. Supplementary file 1D Restype file (in.res). Restype file used in the RosettaDesign calculations. [file elife-47839-supp1.docx]

**Supplementary file 1A. RosettaScripts XML file (.xml)**

<ROSETTASCRIPTS>

<SCOREFXNS>

<hard_symm weights=beta_cst symmetric=1>

<Reweight scoretype=coordinate_constraint weight=0.5 />

<Reweight scoretype=aa_composition weight=1.0 />

</hard_symm>

<soft_symm weights=beta_nov15_soft symmetric=1>

<Reweight scoretype=aa_composition weight=1.0 />

</soft_symm>

<up_ele weights=beta_cst symmetric=1>

<Reweight scoretype=fa_elec weight=1.4 />

<Reweight scoretype=hbond_sc weight=2.0 />

<Reweight scoretype=aa_composition weight=1.0 />

</up_ele>

<hard_symm_cart weights=beta_cart symmetric=1 />

</SCOREFXNS>

<RESIDUE_SELECTORS>

<Index name=res1to16_65to72 resnums=1-16,65-72 />

<Not name=notres1to16_65to72 selector=res1to16_65to72 />

<Index name=res67_68_71 resnums=67,68,71 />

</RESIDUE_SELECTORS>

<TASKOPERATIONS>

<OperateOnResidueSubset name=near_junction selector=notres1to16_65to72 >

<PreventRepackingRLT/>

</OperateOnResidueSubset>

<OperateOnResidueSubset name=mba_binding_res selector=res67_68_71 >

<RestrictToRepackingRLT/>

</OperateOnResidueSubset>

<ReadResfile name=re

<LayerDesign name=all_layers layer=Nterm_core_boundary_surface_Cterm make_pymol_script=0 use_sidechain_neighbors=True core=4 >

<core>

<Helix append="M"/>

</core>

<boundary>

</boundary>

<surface>

</surface>

</LayerDesign>

<LayerDesign name=select_core layer=core make_pymol_script=0 use_sidechain_neighbors=True core=4 />

<LayerDesign name=select_boundary layer=boundary make_pymol_script=0 use_sidechain_neighbors=True core=4 />

<LayerDesign name=select_surface layer=Nterm_surface_Cterm make_pymol_script=0 use_sidechain_neighbors=True core=4 />

<LimitAromaChi2 name=arochi />

<ExtraRotamersGeneric name=ex1_ex2 ex1=1 ex2=1/>

<ExtraRotamersGeneric name=ex1 ex1=1/>

</TASKOPERATIONS>

<FILTERS>

<Geometry name=geom confidence=0 />

</FILTERS>

<MOVERS>

<SetupForSymmetry name=setup_symm definition="/work/sboyken/Rosetta/main/database/symmetry/cyclic/C3_Z.sym"/>

<FastDesign name=fdes scorefxn=hard_symm repeats=1 task_operations=near_junction,mba_binding_res,resin,all_layers,arochi,ex1 >

<MoveMap >

<Span begin=1 end=16 chi=1 bb=1 />

</MoveMap>

</FastDesign>

</MOVERS>

<PROTOCOLS>

<Add mover=setup_symm/>

<Add mover=fdes />

<Add filter=geom />

</PROTOCOLS>

</ROSETTASCRIPTS>

**Supplementary file 1B. Parameter constraint file for amantadine (.cst)**

AtomPair N1 1B N1 1D HARMONIC 0.00 0.01

AtomPair N1 1B N1 1F HARMONIC 0.00 0.01

AtomPair C1 1B C1 1D HARMONIC 0.00 0.01

AtomPair C1 1B C1 1F HARMONIC 0.00 0.01

AtomPair C2 1B C2 1D HARMONIC 2.60 0.01

AtomPair C2 1B C2 1F HARMONIC 2.60 0.01

AtomPair H5 1B H5 1D HARMONIC 2.50 0.01

AtomPair H5 1B H5 1F HARMONIC 2.50 0.01

AtomPair C4 1B C4 1D HARMONIC 2.50 0.01

AtomPair C4 1B C4 1F HARMONIC 2.50 0.01

AtomPair H1 1B N1 1B HARMONIC 1.02 0.01

AtomPair H1 1B C1 1B HARMONIC 2.05 0.01

AtomPair H1 1B C3 1B HARMONIC 2.70 0.01

AtomPair H1 1B H3 1B HARMONIC 2.70 0.01

AtomPair H1 1B H4 1B HARMONIC 3.04 0.01

AtomPair H1 1B C2 1B HARMONIC 4.14 0.01

AtomPair H1 1B H2 1B HARMONIC 4.81 0.01

AtomPair H1 1B C4 1B HARMONIC 4.47 0.01

AtomPair H1 1B H5 1B HARMONIC 5.54 0.01

AtomPair H1 1B H6 1B HARMONIC 4.37 0.01

AtomPair N1 1B C1 1B HARMONIC 1.48 0.01

AtomPair N1 1B C3 1B HARMONIC 2.50 0.01

AtomPair N1 1B H3 1B HARMONIC 2.74 0.01

AtomPair N1 1B H4 1B HARMONIC 2.74 0.01

AtomPair N1 1B C2 1B HARMONIC 3.87 0.01

AtomPair N1 1B H2 1B HARMONIC 4.66 0.01

AtomPair N1 1B C4 1B HARMONIC 4.36 0.01

AtomPair N1 1B H5 1B HARMONIC 5.37 0.01

AtomPair N1 1B H6 1B HARMONIC 4.50 0.01

AtomPair C1 1B C3 1B HARMONIC 1.57 0.01

AtomPair C1 1B H3 1B HARMONIC 2.17 0.01

AtomPair C1 1B H4 1B HARMONIC 2.17 0.01

AtomPair C1 1B C2 1B HARMONIC 2.56 0.01

AtomPair C1 1B H2 1B HARMONIC 3.49 0.01

AtomPair C1 1B C4 1B HARMONIC 3.01 0.01

AtomPair C1 1B H5 1B HARMONIC 3.97 0.01

AtomPair C1 1B H6 1B HARMONIC 3.36 0.01

AtomPair C3 1B H3 1B HARMONIC 1.07 0.01

AtomPair C3 1B H4 1B HARMONIC 1.07 0.01

AtomPair C3 1B C2 1B HARMONIC 1.56 0.01

AtomPair C3 1B H2 1B HARMONIC 2.17 0.01

AtomPair C3 1B C4 1B HARMONIC 2.55 0.01

AtomPair C3 1B H5 1B HARMONIC 3.48 0.01

AtomPair C3 1B H6 1B HARMONIC 2.77 0.01

AtomPair H3 1B H4 1B HARMONIC 1.75 0.01

AtomPair H3 1B C2 1B HARMONIC 2.17 0.01

AtomPair H3 1B H2 1B HARMONIC 2.49 0.01

AtomPair H3 1B C4 1B HARMONIC 2.76 0.01

AtomPair H3 1B H5 1B HARMONIC 3.74 0.01

AtomPair H3 1B H6 1B HARMONIC 2.55 0.01

AtomPair H4 1B C2 1B HARMONIC 2.16 0.01

AtomPair H4 1B H2 1B HARMONIC 2.48 0.01

AtomPair H4 1B C4 1B HARMONIC 3.48 0.01

AtomPair H4 1B H5 1B HARMONIC 4.30 0.01

AtomPair H4 1B H6 1B HARMONIC 3.74 0.01

AtomPair C2 1B H2 1B HARMONIC 1.07 0.01

AtomPair C2 1B C4 1B HARMONIC 1.56 0.01

AtomPair C2 1B H5 1B HARMONIC 2.17 0.01

AtomPair C2 1B H6 1B HARMONIC 2.17 0.01

AtomPair C4 1B H5 1B HARMONIC 1.07 0.01

AtomPair C4 1B H6 1B HARMONIC 1.07 0.01

AtomPair H5 1B H6 1B HARMONIC 1.75 0.01

**Supplementary file 1C. Parameter definition file for amantadine (.params)**

NAME AMA

IO_STRING AMA Z

TYPE LIGAND

AA UNK

ATOM C3 CH2 X -0.00

ATOM C1 VIRT VIRT -0.01

ATOM N1 VIRT VIRT 0.22

ATOM H1 Hpol X 0.20

ATOM C2 CH1 X -0.04

ATOM C4 CH2 X -0.05

ATOM H5 Hapo X 0.03

ATOM H6 Hapo X 0.03

ATOM H2 Hapo X 0.03

ATOM H3 Hapo X 0.03

ATOM H4 Hapo X 0.03

BOND_TYPE N1 C1 1

BOND_TYPE N1 H1 1

BOND_TYPE C1 C3 1

BOND_TYPE C2 C3 1

BOND_TYPE C2 C4 1

BOND_TYPE C2 H2 1

BOND_TYPE C3 H3 1

BOND_TYPE C3 H4 1

BOND_TYPE C4 H5 1

BOND_TYPE C4 H6 1

CHI 1 C3 C1 N1 H1

PROTON_CHI 1 SAMPLES 3 60 -60 180 EXTRA 1 20

CHI 2 C2 C3 C1 N1

CHI 3 C1 C3 C2 C4

NBR_ATOM C3

NBR_RADIUS 4.061247

ICOOR_INTERNAL C3 0.000000 0.000000 0.000000 C3 C1 N1

ICOOR_INTERNAL C1 0.000000 179.999999 1.566251 C3 C1 N1

ICOOR_INTERNAL N1 0.000000 69.171297 1.475135 C1 C3 N1

ICOOR_INTERNAL H1 -59.992968 70.501815 1.019861 N1 C1 C3

ICOOR_INTERNAL C2 -179.913313 69.987429 1.563477 C3 C1 N1

ICOOR_INTERNAL C4 -60.077215 70.557006 1.562665 C2 C3 C1

ICOOR_INTERNAL H5 179.932145 70.486499 1.069869 C4 C2 C3

ICOOR_INTERNAL H6 120.004104 70.545223 1.070033 C4 C2 H5

ICOOR_INTERNAL H2 -120.058942 70.607612 1.069608 C2 C3 C4

ICOOR_INTERNAL H3 -120.092430 70.622215 1.069827 C3 C1 C2

ICOOR_INTERNAL H4 -120.198575 70.836634 1.069942 C3 C1 H3

**Supplementary file 1D. Restype file (in.res)**

ALLAAxc

START

1 A PIKAA SDTN

8 A PIKAA Y
