# Supplementary material for: De novo design of a homo-trimeric amantadine-binding protein
Source: eLife. 2019 Dec 19;8:e47839. doi: 10.7554/eLife.47839 (PMC6922598; doi:10.7554/eLife.47839)
Supplement: Supplementary file 2. — Data collection and refinement statistics for the X-ray structure of ABP Supplementary file 2B Neutron scattering data collection and refinement statistics Data collection and refinement statistics for the neutron and room temperature X-ray structure of ABP. [file elife-47839-supp2.docx]

**Supplementary file 2A. X-ray data collection and refinement statistics**

|  | ABP+amantadine |
| --- | --- |
| **Data collection** |  |
| Space group | P6_3_ |
| Cell dimensions |  |
| *a*, *b*, *c* (Å) | 49.986 49.986 67.339 |
| α, β, γ (°) | 90 90 120 |
| Resolution (Å) | 50.00 - 1.04  (1.06 - 1.04)* |
| *R*_merge_ | 0.056 (0.263) |
| *I* / σ*I* | 62.1 (6.3) |
| Completeness (%) | 99.9 (97.7) |
| Redundancy | 18.9 (7.8) |
|  |  |
| **Refinement** |  |
| Resolution (Å) | 23.431-1.039 |
| No. reflections | 42644 |
| *R*_work_ / *R*_free_ | 19.64/21.39 |
| No. atoms |  |
| Protein | 610 |
| Ligand/ion | 12 |
| Water | 75 |
| *B*-factors | 23.36 |
| Protein | 21.61 |
| Ligand/ion | 16.51 |
| Water | 33.17 |
| R.m.s. deviations |  |
| Bond lengths (Å) | 0.014 |
| Bond angles (°) | 1.35 |

*Values in parentheses are for highest-resolution shell.

**Supplementary file 2B. Neutron scattering data collection and refinement statistics**

Data collection and refinement statistics for the neutron and room temperature X-ray structure of ABP

|  | Neutron | X-ray |
| --- | --- | --- |
| **Data collection** |  |  |
| Space group | P6_3_  50.61,50.61,68.82  90,90,120 | |
| Cell dimensions |  |  |
| *a*, *b*, *c* (Å) |  |  |
| α, β, γ (°) |  |  |
| Resolution (Å) | 36.72 to 2.30 (2.52 to 2.30)* | 68.82 to 1.92 (1.99 to 1.92)* |
| *R*_sym_ or *R*_merge_ | 0.14/0.26 | 0.09 (0.55) |
| *I* / σ*I* | 8.7/1.9 | 23.2 (5.7) |
| Completeness (%) | 73.5 (60.5) | 100 (99.87) |
| Redundancy | 4.0 (2.8) | 17.8 (16.0) |
|  |  |  |
| **Refinement** |  |  |
| Resolution (Å) | 27.06-2.5 | 36.97-1.92 |
| No. reflections | 2639 | 7655 |
| *R*_work_ / *R*_free_ | 27.5/31.1 | 17.4/20.2 |
| No. of non-hydrogen atoms |  |  |
| Protein | 596  11  42  29.16  21.11  41.49  0.019  1.947 | |
| Ligand/ion |  |  |
| Water |  |  |
| *B*-factors |  |  |
| Protein |  |  |
| Ligand/ion |  |  |
| Water |  |  |
| R.m.s. deviations |  |  |
| Bond lengths (Å) |  |  |
| Bond angles (°) |  |  |

*Values in parentheses are for highest-resolution shell.
