# Supplementary material for: De novo design of a homo-trimeric amantadine-binding protein
Source: eLife. 2019 Dec 19;8:e47839. doi: 10.7554/eLife.47839 (PMC6922598; doi:10.7554/eLife.47839)
Supplement: Supplementary file 3. [file elife-47839-supp3.docx]

**Supplementary file 3. NMR line shape fitting analysis with fixed K_D_ values.**

| K_D_ (fixed) | K_off_ (float) | Norm of residuals  (chi-square residuals) |
| --- | --- | --- |
| 12 μM | 38.2 ± 2.8 s^-1^ | 8436.49 |
| 24 μM | 60.9 ± 5.6 s^-1^ | 8384.08 |
| 48 μM | 119.0 ± 6.7 s^-1^ | 8455.18 |
